# Efficacy of low-intensity pulsed ultrasound for the treatment of viral pneumonia: study protocol for a randomized controlled trial

**DOI:** 10.1186/s13063-023-07382-1

**Published:** 2023-06-09

**Authors:** Xiao Li, Wen Li, Lianjie Sun, Junyi Ren, Ying Xu, Yuanyi Zheng, Wenkun Bai

**Affiliations:** 1 Department of Ultrasound in Medicine, Shanghai Sixth People’s Hospital Affiliated to Shanghai Jiao Tong University School of Medicine, Shanghai Institute of Ultrasound in Medicine, Yishan Road 600, Shanghai, 200233 China; 2 Department of Cardiovascular Surgery, The Affiliated Hospital of Qingdao University, Wutaishan Road 1677, Qingdao, 266555 China

**Keywords:** Viral pneumonia, Ultrasonic therapy, Ultrasonic waves, LIPUS, Inflammation

## Abstract

**Background:**

Viral pneumonia has always been a problem faced by clinicians because of its insidious onset, strong infectivity, and lack of effective drugs. Patients with advanced age or underlying diseases may experience more severe symptoms and are prone to severe ventilation dysfunction. Reducing pulmonary inflammation and improving clinical symptoms is the focus of current treatment. Low-intensity pulsed ultrasound (LIPUS) can mitigate inflammation and inhibit edema formation. We aimed to investigate the efficacy of therapeutic LIPUS in improving lung inflammation in hospitalized patients with viral pneumonia.

**Methods:**

Sixty eligible participants with clinically confirmed viral pneumonia will be assigned to either (1) intervention group (LIPUS stimulus), (2) control group (null stimulus), or (3) self-control group (LIPUS stimulated areas versus non-stimulated areas). The primary outcome will be the difference in the extent of absorption and dissipation of lung inflammation on computed tomography. Secondary outcomes include changes in lung inflammation on ultrasonography images, pulmonary function, blood gas analysis, fingertip arterial oxygen saturation, serum inflammatory factor levels, the sputum excretion volume, time to the disappearance of pulmonary rales, pneumonia status score, and course of pneumonia. Adverse events will be recorded.

**Discussion:**

This study is the first clinical study of the efficacy of therapeutic LIPUS in the treatment of viral pneumonia. Given that the current clinical recovery mainly depends on the body’s self-limiting and conventional symptomatic treatment, LIPUS, as a new therapy method, might be a major advance in the treatment of viral pneumonia.

**Trial registration:**

ChiCTR2200059550 Chinese Clinical Trial Registry, May 3, 2022.

**Supplementary Information:**

The online version contains supplementary material available at 10.1186/s13063-023-07382-1.

## Introduction

### Background and rationale {6a}

Viral pneumonia is a disease in which viral infection of the upper respiratory tract spreads downward, causing pulmonary inflammation and gas exchange abnormality, which is characterized by insidious onset and strong infectivity [1]. Most of the virus infections are mild to moderate and the clinical manifestations are mainly fever, headache, dry cough, and generalized myalgia. Other viruses can infiltrate the lungs and cause viral pneumonia. In recent years, some newly discovered cases of severe pneumonia caused by viruses have attracted more and more global attention, such as highly pathogenic avian influenza, adenovirus pneumonia, and novel coronavirus pneumonia, which are more serious among the elderly, obese, and patients with underlying diseases.

The treatment of viral pneumonia has always been a problem for clinicians. Due to the high variability of most viruses, the development of antiviral drugs faces great challenges. At present, symptomatic support treatment is the mainstay of clinical management.

Low-intensity pulsed ultrasound (LIPUS), a therapeutic ultrasound technique that has emerged in recent years [2], has been used to treat soft tissue and musculoskeletal injuries [3–6]. Studies have found that LIPUS can not only exert non-invasive physical stimulation through low-intensity delivery in the mode of pulsed waves but also can carry out biological therapy, which helps to mitigate inflammation, facilitate tissue repair, inhibit edema formation, and stimulate angiogenesis [7]. At present, therapeutic LIPUS has been experimentally or clinically studied in many diseases and achieved good efficacy [8–11].

Overactivated inflammatory response and abnormal immunologic state in patients with viral pneumonia were significantly associated with a high risk of severe cases [12, 13]. Given that LIPUS can up-regulate the expression of anti-inflammatory genes, upregulate immunosuppressive cell regulators and anti-inflammatory cytokines [14], and play a good role in mitigating inflammation, we tried to use wearable LIPUS to irradiate lung inflammation to improve clinical symptoms and shorten the disease course. At the same time, the physical effect of LIPUS can promote the loosening of the sputum scab attached to the inner wall of the small bronchi through low-frequency impact force [15, 16] and promote the discharge of sputum in the small airway. The mechanical and cavitation effect of ultrasound can also destroy the thrombus surface, expose the thrombolytic site, and promote the combination of thrombolytic drugs with fibrin to promote thrombolysis [17], thereby improving pulmonary blood circulation and preventing venous stasis.

Therefore, we propose a strategy of LIPUS treatment program for patients with viral pneumonia. We aim to conduct a randomized double-blind controlled clinical superiority trial that could evaluate the efficacy of LIPUS on hospitalized patients with viral pneumonia.

### Objectives {7}

#### Primary objective

The primary objective is to examine whether LIPUS therapy is more effective to mitigate lung inflammation in viral pneumonia patients than conventional symptomatic care control.

#### Secondary objectives

1. To determine whether there is a difference in pulmonary function between LIPUS treatment group and control group
2. To determine whether there is a difference in arterial oxygen saturation and serum inflammatory factor levels between LIPUS treatment group and control group
3. To determine whether there is a difference in the improvement of clinical symptoms among patients

### Trial design {8}

**Fig. 1:**
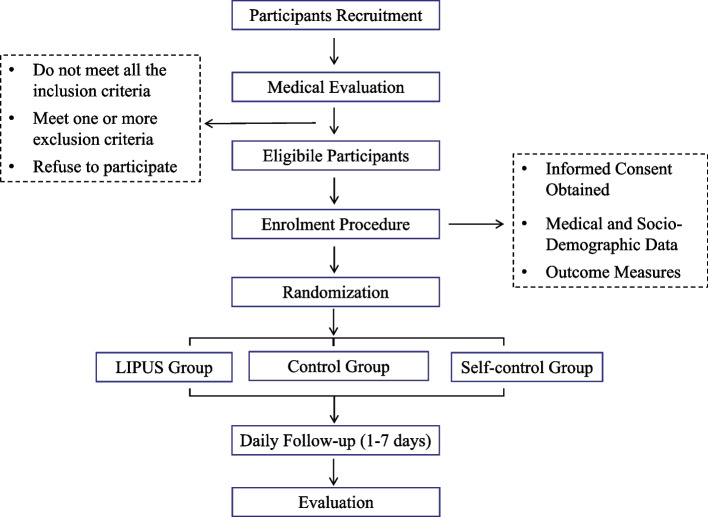
Participant flow chart. LIPUS, low-intensity pulsed ultrasound

The study design is a randomized, double-blind, controlled clinical superiority trial. Participants will be randomized into three groups in a 1:1:1 allocation ratio: (1) the intervention group (LIPUS stimulus), (2) the control group (null stimulus), or (3) the self-control group (stimulated vs non-stimulated areas). Treatment is expected to last 7 days, and participant recruitment and data collection will be completed within 12 months. The study protocol flow chart is described in Fig. 1.

## Methods: participants, interventions, and outcomes

### Study setting {9}

This is a single-center clinical study, which will be carried out in the Lingang Campus of Shanghai Sixth People’s Hospital Affiliated to Shanghai Jiao Tong University School of Medicine.

### Eligibility criteria {10}

Eligible participants will undergo laboratory and imaging examinations to confirm the diagnosis of viral pneumonia and to assess eligibility to participate in the study.

Patients who meet all the following enrollment criteria will enter this study after signing the informed consent: (1) age 18–80 years old; (2) hospitalized patients had pneumonia-related symptoms, computed tomography (CT) examination met the diagnostic criteria of pneumonia, and the etiology examination suggested a viral origin; (3) patients who can cooperate with various examinations during clinical research; (4) the patient participates voluntarily, and he/she or his/her family agrees and signs the informed consent.

Patients who meet the following criteria will be excluded: (1) other types of pneumonia, such as bacterial pneumonia, mycoplasma pneumonia, falling accumulation pneumonia, etc.; (2) patients with bronchiectasis or lung malignancies; (3) patients with chest skin injury who cannot wear the LIPUS device; (4) pregnant and breastfeeding women; (5) contraindications to the therapeutic ultrasonography (US), including dermatological conditions, local abnormal sensations in the chest or back, epilepsy, etc.; (6) patients with mental illness or unable to communicate normally with staff.

### Who will take informed consent? {26a}

The research assistant (X.L.) will take informed consent before trial baseline evaluation in the participant reception room. Caregivers will give informed consent to potential participants before the time point of allocation. Participants will be notified why the study is conducted, what they will be doing, and the possible benefits and risks. If participants have any questions, they are free to ask them, and they can decide whether to participate in the study after fully understanding.

### Additional consent provisions for collection and use of participant data and biological specimens {26b}

No additional biological samples were collected for the study. No subject data are currently available for future studies. Additional consent from participants will be obtained if relevant data from participants is required in future studies.

## Interventions

### Explanation for the choice of comparators {6b}

The usual practice combined with empty stimulation (a placebo effect) was selected as the comparator condition in order to detect whether the anticipated benefit of LIPUS treatment would produce better anti-inflammatory effect than the current conventional symptomatic care. In addition, a self-control group (LIPUS stimulated areas versus non-stimulated areas) was established to eliminate the interference of individual differences on the results.

### Intervention description {11a}

All participants will participate in a health education of approximately 20 min led by the research assistant prior to intervention treatment. The content of the education includes the transmission route of viruses, preventive measures, clinical manifestations, general treatment measures, etc. It is recommended that patients maintain hygiene, exercise moderately, drink an appropriate amount of water, and have a light diet.

Participants in the intervention group will be treated with wearable LIPUS therapeutic apparatus (UT – HP 05 21, Shanghai, China) for 7 days at a frequency of 572 kHz, an intensity of 820 mW/cm^2^, and a duty cycle of 50% (1 s inter-stimulus interval). The apparatus consists of two movable ultrasound probes and a central device. The operator fixed the ultrasound probes on the patient’s chest or back and adjusted the position of the probes according to the shape and scope of the lung lesion to perform vertical or parallel sonication therapy (Supplementary Fig. 1). During hospitalization, patients received 30-min treatments at 8:00 am and 2:00 pm daily.

Participants in the control group will be given a “null stimulus,” that is, the LIPUS therapeutic apparatus will be worn but the sonication treatment will not be started. These patients will be treated at the same time of day as the patients in the test group, but neither the patients nor the operators of the LIPUS equipment are aware of the grouping.

Participants in the self-control group will be compared the LIPUS-treated area with the non-treated area in the lungs. First, a CT examination is performed to locate the body surface of the inflammatory exudative area. If the patient has bilateral pulmonary exudative inflammation, LIPUS treatment will be performed on one side of the lung, and the other will serve as the self-control group. Limited by the frequency of LIPUS, ultrasound cannot penetrate through one lung tissue to the other, and its maximum penetration depth is limited to the inside of the target lung. If the patient has unilateral pulmonary exudative inflammation, the inflammatory area will be divided into the upper and lower areas, and a single-area stimulus will be performed. The diameter of the two ultrasound probes is approximately equal to the width of the intercostal space, and the probes can be placed in the same intercostal space or in different intercostal spaces. The direction of sound beam can be adjusted manually. Limited by the width of the sound beam and the plane of sonication, the non-target inflammatory area above or below can be considered not to be affected.

### Criteria for discontinuing or modifying allocated interventions {11b}

Patients are free to withdraw from participation in the study at any time upon request, without any consequence. If a participant chooses to discontinue the study intervention, we will not collect or use their data.

LIPUS is an FDA-approved and safe treatment modality. To date, no adverse events have been reported with the low-power LIPUS regimen in soft tissue. Therefore, we anticipate that the risk to the safety of participants will be minimal. However, nursing staff will still monitor potential mild adverse reactions such as mild local swelling, spotting bleeding, enhanced local pain response, and local hyperesthesia or decrease. In case of corresponding symptoms, appropriate symptomatic treatment shall be carried out. Regardless, participants can withdraw from the study at any time if they experience adverse events that are unacceptable to them.

### Strategies to improve adherence to interventions {11c}

Lung CT examinations, ultrasound examinations, trial-related laboratory examinations, and relevant symptomatic treatment measures received by patients before and after treatment are provided free of charge to improve adherence to interventions.

### Relevant concomitant care permitted or prohibited during the trial {11d}

All participants will be given routine nutritional support care. We discourage additional treatments to that assigned (that is, not per protocol) during the intervention period. Participants will report all not-per-protocol treatments, such as adding antiviral agents.

### Provisions for post-trial care {30}

Trial interventions are designed to inflict minimal to no harm. If a participant suffers any injury due to participating in the trial, we will evaluate, document, and provide appropriate medical care and pay for all related medical expenses.

## Outcomes {12}

### Primary outcome

The primary outcome measure will be the difference in the extent of absorption and dissipation of lung inflammation on CT images. Result comparisons will be made between the test group and the control group and between the LIPUS-treated and non-treated areas within the self-control group.

Pulmonary inflammation on CT images will be assessed by an expert chest radiologist before and 7 days after treatment to compare the area and volume of inflammation in the target area of the lung, as well as the area absorption rate (AAR) or volume absorption rate (VAR). A Pair software will be used to mark and 3D reconstruct the target area of CT images and record the data of pulmonary inflammation changes. AAR is defined as [(target site inflammation area before treatment − target site inflammation area after treatment)/target site inflammation area before treatment] × 100 (%). VAR is defined as [(target site inflammation volume before treatment − target site inflammation volume after treatment)/target site inflammation volume before treatment] × 100 (%).

### Secondary outcomes

Secondary outcome measures will be differences in the extent of lung inflammation dissipation on US images, pulmonary function, blood gas analysis, fingertip arterial oxygen saturation (SaO_2_) monitoring, serum inflammatory factor levels, the sputum excretion volume, time to the disappearance of pulmonary rales, pneumonia status score, and course of pneumonia.

#### Lung ultrasound scoring system

US will be performed before and 7 days after treatment by sonographers with certified competency in thoracic US, blind to CT findings. Chest scans are performed using the convex 3.5–5 MHz and linear 4–8 MHz probes of the US equipment, with the patient in the sitting position. According to previous studies [18], each hemithorax was divided into 4 regions: anterior-lateral sector, posterior sector, upper halve of the third intercostal space, and lower half of the third intercostal space. The evaluation criteria for pulmonary severity classification will be based on the scoring system proposed by Soldati et al. [19] (0 = regular pleural line, A-lines present; 1 = indented pleural line, focal B lines; 2 = broken pleural line, subpleural consolidations; and 3 = white lung with or without consolidations).

#### Pulmonary function

Pulmonary function tester will detect vital capacity (VC), forced vital capacity (FVC), forced expiratory volume in one second (FEV_1_), peak expiratory flow (PEF), and maximal mid-expiratory flow (MMEF) before and 7 days after treatment.

#### Blood gas analysis and fingertip SaO_2_ monitoring

The blood gas analyzer will detect the arterial blood partial pressure of oxygen (PaO_2_), arterial blood carbon dioxide partial pressure (PaCO_2_), SaO_2_, and the pH value before and 7 days after treatment. The fingertip photoelectric sensor will also be used to measure the SaO_2_, and the nursing staff will record the data before treatment, 2 h after daily treatment, and after the total course of treatment.

#### Serum inflammatory factor levels

Before and 7 days after treatment, nurses will draw 5 ml of fasting venous blood from patients in the morning and detect the proportion of inflammatory cells and the levels of inflammatory factors such as C-reactive protein (CRP), tumor necrosis factor (TNF)-α, interleukin (IL)-1, IL-2, IL-6, and IL-8, and procalcitonin (PCT) in peripheral blood.

#### The sputum excretion volume

For patients who can cough spontaneously, nurses will collect and record the amount of sputum that can be excreted in 24 h, and those with negative pressure suction will use a disposable sputum suction device to record the amount of sputum excreted after each treatment. Collection by trained nurses takes place daily at 8:00 am and ends at 8:00 am the next day. Less than 10 ml/24 h is a small amount of sputum, 10–150 ml/24 h is a moderate amount of sputum, and more than 150 ml/24 h is a large amount of sputum. The color and consistency of the sputum will also be recorded.

#### Time to the disappearance of pulmonary rales

Auscultation of pulmonary rales will be performed by an experienced respiratory physician before the treatment, 2 h after the daily treatment period, and at the end of the treatment without using auxiliary expectoration equipment.

#### Pneumonia status score

The pneumonia severity index (PSI) [20] grading and clinical pulmonary infection score (CPIS) [21] will be used for evaluation before treatment and after 7 days of treatment, and the worst value within 24 h was calculated on the 7th day. The PSI risk level is reduced by two levels to be markedly effective, reduced by one level is effective, and the level is not reduced or increased is invalid. The clinical therapeutic effects of the test group and the control group will be compared by (markedly effective + effective)/total number of cases × 100%.

#### The course of pneumonia

The hospitalization time and the time from the first symptom to discharge of the patients in each group will be counted. The discharge criteria for confirmed patients are as follows: (1) body temperature returned to normal for more than 3 days, (2) respiratory symptoms improved significantly, (3) pulmonary imaging showed significant improvement in acute exudative lesions, and (4) two consecutive viral antigen or nucleic acid tests were negative.

### Participant timeline {13}

**Fig. 2:**
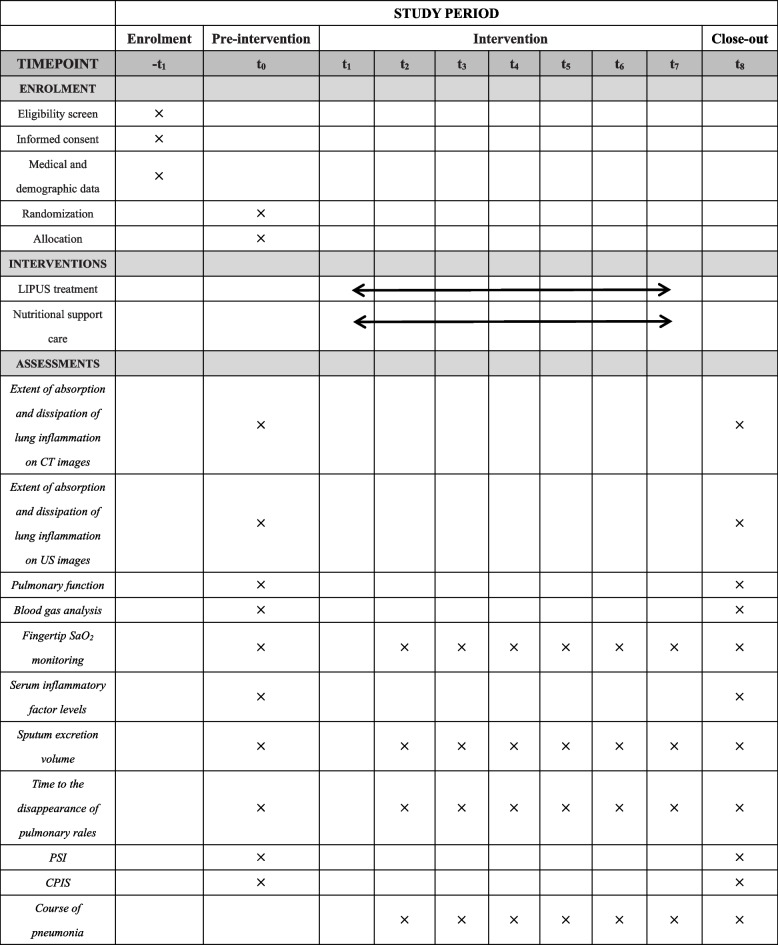
Schedule of enrollment, interventions, and assessments (SPIRIT figure). LIPUS, low-intensity pulsed ultrasound; CT, computed tomography; US, ultrasonography; SaO_2_, arterial oxygen saturation; PSI, pneumonia severity index; CPIS, clinical pulmonary infection score

The participant timeline is shown in Fig. 2.

### Sample size {14}

Sample size and power calculation are based on the primary outcomes. All sample size calculations assume two-sided analysis with a power of 90% (1-*β* = 0.90) at a significant level of *α* = 0.05. The use of therapeutic LIPUS in viral pneumonia has not yet been reported. Based on the time span of this clinical trial and the number of patients expected to meet the inclusion criteria in our hospital, it is estimated that a total of 66 participants will be required (1:1:1 allocation ratio). We anticipate minimal missing data (< 5%) for the primary outcome (day 7 pulmonary inflammation on CT images) because participants were all inpatients and we have daily contact with them during the treatment phase. With a dropout rate of approximately 5%, a final sample size of 70 participants is required.

### Recruitment {15}

Participants will be recruited from the Lingang Campus of Shanghai Sixth People’s Hospital Affiliated to Shanghai Jiaotong University School of Medicine, and they are all inpatients who have undergone laboratory and imaging examinations to confirm the diagnosis of viral pneumonia. The doctors will contact with the patients and their families, and after obtaining the informed consent from the patients themselves or their family members, doctors will conduct screening according to the inclusion and exclusion criteria. Only those who meet all recruitment requirements and do not meet any exclusion criteria will participate in this study.

## Assignment of interventions: allocation

### Sequence generation {16a} and concealment mechanism {16b}

Participants will be randomized into three groups (LIPUS group; control group; self-control group) in a ratio of 1:1:1 using a computer-generated randomization sequence. Research assistants not involved in clinical care or outcome assessment will prepare sequentially numbered, opaque, sealed envelopes based on the randomization list and ensure the confidentiality and independence of the allocation data. When appropriate, the assistant will open envelopes and ensure the coordination of therapeutic interventions.

### Implementation {16c}

The sealed envelopes will be opened and reconcealed during the first LIPUS stage, before intervention by the staff responsible for randomization. The code will then be notified to the LIPUS experimenter.

## Assignment of interventions: blinding

### Who will be blinded {17a}

Both patients and clinic staffs (caregivers, outcome assessors, and statistical analysts, etc.) will remain blind to group allocation. Whether the intervention is LIPUS-treated or non-stimulated will not be revealed throughout the study.

The clinician (W.L.) who performed LIPUS therapy was not blinded to the grouping results and the course of treatment. The clinicians, Y.Z. and W.B., have access to the final trial dataset.

### Procedure for unblinding if needed {17b}

Once the analysis of the primary and secondary outcomes is complete, the identity of the three groups will be revealed.

## Data collection and management

### Plans for assessment and collection of outcomes {18a}

The primary outcome data will be collected at the baseline and the termination of treatment; the secondary outcomes will be collected at pre-randomization, a fixed time every day and/or after 7 days of treatment. All variables in the protocol will be documented in an encrypted Microsoft Office Excel. The investigators who enter information into the office excel are responsible for ensuring the accuracy and completeness of information. A day-to-day management group, consisting of the principal investigator, a doctoral student, a research coordinator and an assistant manager, conducts clinical monitoring activities.

### Plans to promote participant retention and complete follow-up {18b}

The patients will complete all evaluations and treatments during hospitalization. Lung CT examinations, ultrasound examinations, and trial-related laboratory examinations received by patients before and after treatment are provided free of charge. Once a patient withdraws from the study during treatment (does not complete the full 7-day treatment period), their data will not be retained. Every effort should be made to encourage patients to remain in the study for the duration of their planned outcome assessments.

### Data management {19}

Investigators will collect data on participants’ baseline status and daily after randomization. All data obtained will be stored electronically in a securely accessible database and kept strictly confidential. The data transfer process will encrypt and any personally identifiable information will be deleted. De-identification of the data for analysis was allowed only during the final phase of this trial.

Registered participants will be withdrawn from the study if (1) the participant withdraws his/her consent and (2) the exclusion criteria is discovered after registration. The date and reason of the suspension will be recorded, and their data will not be retained.

### Confidentiality {27}

All personal data of registered participants will be assigned a unique identifier and stored on a secure server available only to researchers with administrative privileges. After the trial, only non-personal data will be available for analysis in the data repository.

### Plans for collection, laboratory evaluation, and storage of biological specimens for genetic or molecular analysis in this trial/future use {33}

Not applicable; no biological specimens will be collected.

## Statistical methods

### Statistical methods for primary and secondary outcomes {20a}

Analysis of baseline characteristics of the three groups will be summarized by appropriate descriptive statistics. Analysis of both the primary and secondary outcomes will be performed blinded to treatment assignment and analyzed on the intention-to-treat approach [22], with all randomized participants retaining their original randomization group. If these missing data are judged to be random, multiple imputations of the chained equation will be used to address missing data caused by lost access and non-response.

Normally distributed data were expressed as the mean (standard deviation), whereas data not normally distributed was expressed as the median (interquartile range). The independent sample test was used for the comparison between groups, and the paired sample test was used for the comparison within the group before and after treatment. For clinical symptoms, variables were expressed as percentages. A *χ*^2^ test or Fisher’s exact test was used to compare categorical data. The conventional level of 0.05 for statistical significance was adopted to evaluate the *p*-values. Statistical analysis was carried out by using SPSS software, version 26.

### Interim analyses {21b}

No interim analyses will be planned.

### Methods for additional analyses (e.g., subgroup analyses) {20b}

No any subgroup analysis will be conducted.

### Methods in analysis to handle protocol non-adherence and any statistical methods to handle missing data {20c}

We anticipate minimal missing data (< 5%) for the primary outcome (day 7 pulmonary inflammation on CT images) because participants were all inpatients and we have daily contact with them during the treatment phase. We will evaluate the impact of missing data by calculating the difference across plausible and extreme missing data scenarios. If our primary test is significant, we will also calculate the size of the interaction between the group and missingness required to make this difference non-significant. If 10% or more of the baseline samples are excluded due to missing data, we will use multiple imputation techniques for analysis.

### Plans to give access to the full protocol, participant-level data, and statistical code {31c}

Anonymized participant data may be shared from the corresponding author on reasonable request. There are no intentions to grant public access to participant data and statistical code.

## Oversight and monitoring

### Composition of the coordinating center and trial steering committee {5d}

The clinical monitoring activities will be performed by a day-to-day management group consisting of the principal investigator (PI), a doctoral student, a research coordinator, and an assistant manager. The group will meet weekly. This trial does not have a trial steering committee.

### Composition of the data monitoring committee, its role and reporting structure {21a}

No formal data monitoring committee has been established for this trial, as there are no interim analyses and no planned procedures for early stopping.

### Adverse events reporting and harms {22}

All adverse events, which refer to any negative or undesired reaction to the intervention, will be recorded through the patient-reported symptoms and observations by the researcher at each visit. Lung injury and hemorrhage, possibly caused by the US, have been reported in the past with acoustic pressure levels of at least 1 MPa, much higher than the LIPUS parameters proposed here. And to date, no adverse events have been reported with the low-power LIPUS regimen in soft tissue. Potential mild adverse reactions such as mild local swelling, spotting bleeding, enhanced local pain response, and local hyperesthesia or decrease. In case of corresponding symptoms, appropriate symptomatic treatment shall be carried out.

### Frequency and plans for auditing trial conduct {23}

Not applicable. No auditing is planned in this trial. The PIs will meet weekly during the study period to review trial progress.

### Plans for communicating important protocol amendments to relevant parties (e.g., trial participants, ethical committees) {25}

Before making any protocol modifications, approval must be obtained from the Ethics Committee of Shanghai Sixth People’s Hospital. If approved, these modifications must be reported in the trial register and included in the final report of research data.

### Dissemination plans {31a}

Clinical trial results are expected to be published in scientific medical journals and national and international conferences. The first and corresponding author will hold primary responsibility for the publication of the results of this clinical trial.

## Discussion

The pathological mechanism of viral pneumonia mainly involves a series of “cytokine storms,” tissue edema, vascular endothelial damage, increased vascular permeability, and thrombosis, which leads to a series of clinical symptoms, and may occur dyspnea or impaired oxygenation in severe cases [23, 24]. At present, the focus of treatment for diagnosed patients is symptomatic and we try to use the mechanical and biological effects of LIPUS for treatment.

On the one hand, specific ultrasound intensity, frequency, pulse width, and duration are used for non-invasive physical therapy, and on the other hand, the anti-inflammatory, anti-thrombotic, and pro-reparative effects of LIPUS are combined to relieve the lung condition and reduce the clinical symptoms of patients. Mitogen-activated protein kinase (MAPK) and nuclear factor-kappa B (NF-κB) are classical inflammatory response pathways. A large number of studies have found that the above inflammatory pathways are significantly inhibited after LIPUS irradiation and inflammatory cytokines such as TNF-α, IL-1β, and IL-6 decreased accordingly [25, 26]. The potential of LIPUS to regulate the phenotype of inflammatory cells and reduce the number of neutrophils and inflammatory macrophages was also observed in the musculoskeletal injury model [27, 28].

In this study, we randomly divided patients with viral pneumonia into three groups and evaluated the efficacy of LIPUS by comparing the pulmonary inflammatory state after treatment with the control group and the self-control group. In addition to the control group, we also set up a self-control group to better eliminate the impact of individual differences on the results. If the research results suggest that the curative effect of the LIPUS treatment group is significantly better than that of the control group and the self-control group, it indicates that the LIPUS treatment is effective; if the curative effect of the LIPUS group is better than that of the control group, but there is no difference with the self-control group, it indicates that the LIPUS intervention effect is fair. The statistical difference may be due to individual differences, and the sample size needs to be further expanded for evaluation; if the curative effect of the LIPUS group is not statistically different from that of the control group and the self-control group, LIPUS therapy can be considered poor.

To date, no adverse events have been reported during LIPUS treatment. However, the exact biological effects of LIPUS in tissues are not fully understood. Although LIPUS has been investigated in many therapeutic applications experimentally or clinically and achieved good curative effects [29–31], further research is still needed to fully understand the exact mechanism in humans.

## Strengths and limitations

We proposed and demonstrated a novel approach to promote inflammatory uptake in viral pneumonia. Given that the current clinical recovery mainly depends on the body’s self-limiting and conventional symptomatic treatment, LIPUS, as a new therapy method, might be a major advance in the treatment of viral pneumonia. The clinical application of wearable and easily operative LIPUS without radiation and side effects may be more extensive than limited to viral pneumonia. Combined with the therapeutic mechanism, it is speculated that LIPUS may also play a certain role in the treatment of other types of pneumonia. And it should also have advantages in pediatric patients and home treatment.

This study has two main limitations. First, we only compared the difference between LIPUS treatment and non-treatment and did not compare LIPUS with commonly used clinical anti-inflammatory drugs, immunomodulatory drugs, or anticoagulation, which made the observation of clinical efficacy not comprehensive enough. Second, this is a single-center study. Due to the limited number of wearable US devices, it has not been further developed in other research centers.

To the best of our knowledge, this study is the first clinical study of the efficacy of therapeutic LIPUS in the treatment of viral pneumonia. Compared with drug treatment or other artificial/mechanical assisted treatment equipment, a wearable LIPUS device will be simpler, safer, and more comprehensive and is expected to greatly improve the continuity and compliance of clinical treatment for patients with viral pneumonia.

## Trial status

The first version of the protocol was approved by the institutional ethical committee on June 6, 2022, and this protocol is version 1.0. The design and improvement of the chest wearable LIPUS therapeutic device, as well as the confirmation of ultrasonic stimulation parameters, are currently underway. Patient recruitment and data collection will be carried out in our hospital from October 2022 to October 2023, and data will be collated and analyzed later.

## Supplementary Information

**Additional file 1: Supplementary Fig. 1.** Schematic diagram of wearable LIPUS therapeutic instrument.

## Data Availability

The datasets generated and analyzed during the current study are not publicly available because the protocol has not been completed at the time of submission but will be available from the corresponding author upon reasonable request.
