# Supplementary figures and images for: Efficacy of low-intensity pulsed ultrasound for the treatment of viral pneumonia: study protocol for a randomized controlled trial
Source: Trials. 2023 Jun 9;24:389. doi: 10.1186/s13063-023-07382-1 (PMC10250850; doi:10.1186/s13063-023-07382-1)

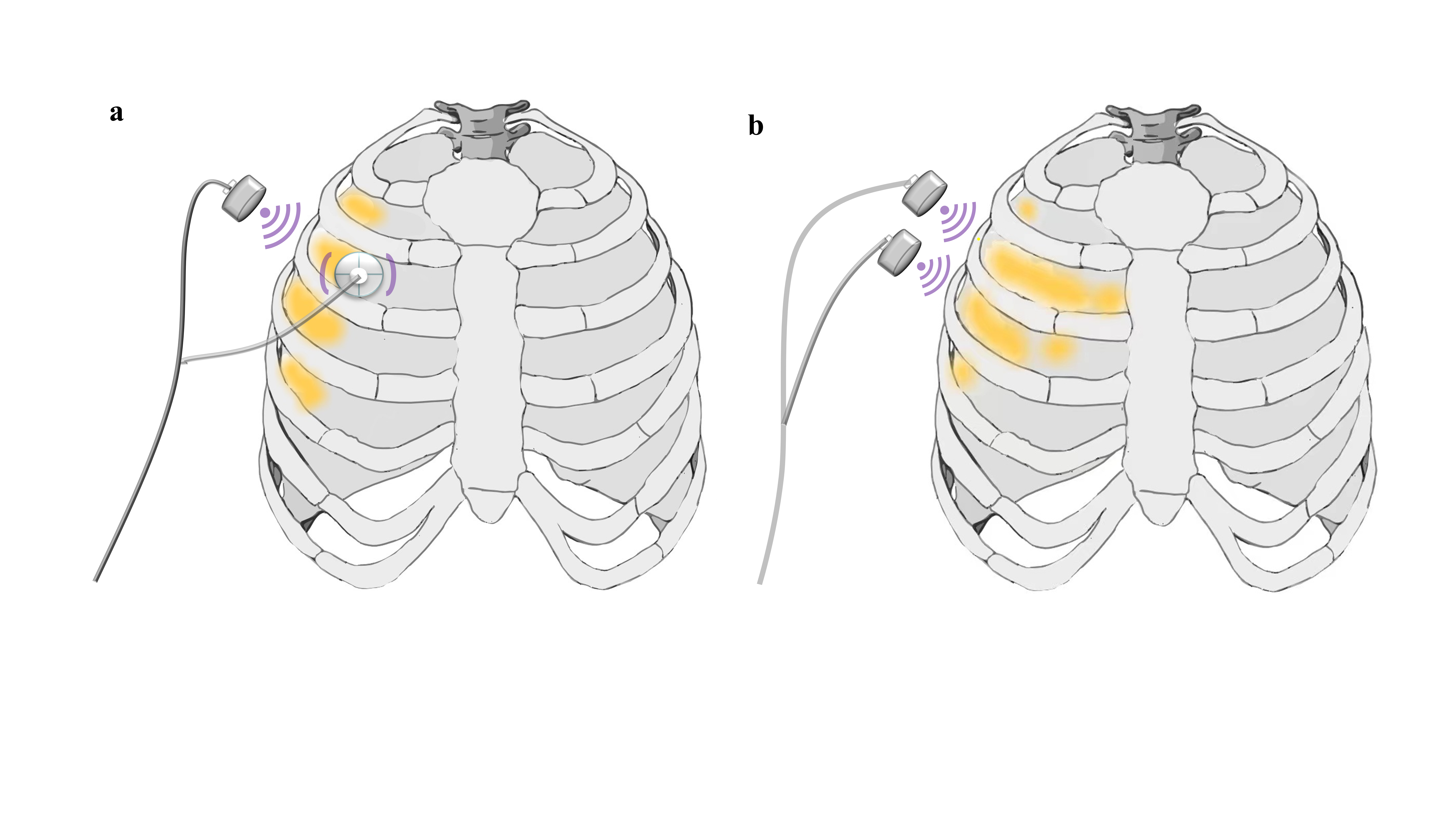

Supplement: Supplementary file 1 — Additional file 1: Supplementary Fig. 1. Schematic diagram of wearable LIPUS therapeutic instrument. [file 13063_2023_7382_MOESM1_ESM.tif]
